# Impact of Digital Smile Design on Patient Satisfaction and Treatment Acceptance in Esthetic Dental Practice: A Clinical Study

**DOI:** 10.7759/cureus.106350

**Published:** 2026-04-03

**Authors:** Syed Yasir Qadiri, Malik Mahmud Iqbal Hossain, Bushra Irum, Aasima Ishaq

**Affiliations:** 1 Department of Restorative Dentistry, College of Dentistry, Najran University, Najran, SAU; 2 Department of Conservative Dentistry and Endodontics, Private Practice, AI Dental Clinic, Anantnag, IND

**Keywords:** design, digital, esthetics, patient satisfaction, smile

## Abstract

Introduction: Esthetic dentistry increasingly emphasizes patient-centered treatment planning and effective communication between clinicians and patients. Conventional consultation methods often rely on verbal explanations and static images, which may not adequately convey the expected esthetic outcomes. Digital Smile Design (DSD) has emerged as a modern digital tool that allows clinicians to simulate potential smile outcomes. This digital approach may improve patient understanding, enhance confidence in treatment planning, and increase patient satisfaction with the consultation process. This study aimed to evaluate the impact of DSD on patient satisfaction and treatment acceptance during esthetic dental consultation.

Materials and methods: This prospective clinical study was conducted in the Department of Restorative Dentistry and included 80 patients who sought esthetic dental treatment. Following a conventional consultation involving a clinical examination and verbal explanation of the treatment plan, patient perceptions were recorded. A digital smile simulation was then generated using the Smile Designer Pro software (Tasty Tech Ltd., London, United Kingdom) and presented to the patients. Patient understanding of the treatment plan and confidence in the proposed treatment were assessed before and after the digital smile simulation using a 5-point Likert scale. Overall satisfaction with the consultation process was evaluated using a 10-point Visual Analog Scale (VAS). Statistical analysis was performed using IBM SPSS Statistics for Windows, Version 20 (Released 2015; IBM Corp., Armonk, New York). Descriptive statistics were calculated, and pre- and post-consultation scores were compared using a paired t-test with a significance level set at p < 0.05.

Results: A significant improvement in patient understanding and confidence was observed following the DSD consultation. Scores related to the clarity of procedures, understanding of treatment duration, costs, and limitations increased significantly after digital visualization (p < 0.001). Patient confidence in the anticipated final smile, confidence in the dentist, certainty of proceeding with treatment, and ability to visualize the expected outcome also improved significantly (p < 0.001). Overall, satisfaction with the consultation process was high, with the majority of participants reporting improved communication and greater involvement in treatment planning. In addition, 85% of the patients accepted the proposed treatment following digital smile simulation.

Conclusion: DSD significantly enhanced patient understanding, confidence, satisfaction, and treatment acceptance during esthetic dental consultation. Its integration into clinical practice may improve communication and promote predictable patient-centered treatment planning in esthetic dentistry.

## Introduction

Esthetic dentistry has gained considerable importance in modern dental practice, as patients increasingly seek treatments that enhance the appearance of their smiles. A harmonious smile is influenced by several factors, including tooth shape, size, alignment, gingival display, and facial proportions [1,2]. Traditionally, treatment planning in esthetic dentistry relies on diagnostic casts, clinical photographs, and the clinician’s subjective interpretation to communicate the expected treatment outcome to the patients [3]. However, these conventional methods often present limitations in effectively conveying the final esthetic result, which may lead to misunderstandings, unrealistic expectations, or reduced patient confidence in the proposed treatment plan.

Digital Smile Design (DSD) has emerged as an innovative tool that facilitates a more predictable and patient-centered approach to esthetic treatment planning. Using digital software, clinicians can simulate the proposed smile outcome and visually demonstrate potential changes in tooth form, alignment, and overall smile harmony [4,5]. This digital visualization enhances communication between the dentist and the patient, allowing individuals to better understand the proposed treatment and actively participate in the decision-making process [4]. Consequently, DSD is increasingly being incorporated into routine clinical practice to improve treatment planning, patient education, and overall satisfaction.

Despite the growing popularity of DSD, there is still limited clinical evidence evaluating its direct impact on patient satisfaction and acceptance of esthetic dental treatments, with controversial results [4,6]. Understanding the role of digital visualization in improving patient perception and confidence in treatment planning is important for optimizing clinical outcomes and enhancing the patient experience. Therefore, assessing patient-reported outcomes following DSD use may provide valuable insights into its effectiveness as a communication and planning tool in esthetic dentistry.

This study aimed to evaluate the impact of DSD on patient satisfaction and acceptance of esthetic dental treatment. The objectives were to assess patient understanding of the proposed treatment plan, evaluate the level of patient confidence following digital smile simulation, and determine the overall patient satisfaction with the DSD-assisted consultation process.

## Materials and methods

This prospective clinical study was conducted in the Department of Restorative Dentistry, College of Dentistry, Najran University, Saudi Arabia, to evaluate the impact of a DSD on patient satisfaction during esthetic dental treatment planning. Ethical approval for the study was obtained from the Institutional Ethics Committee (202601-07-041945-099128) prior to the commencement of the study, and written informed consent was obtained from all participants after explaining the nature and purpose of the study.

The sample size was calculated using G*Power software (version 3.1; Heinrich Heine University, Düsseldorf, Germany) based on a paired t-test comparing patient satisfaction scores before and after the DSD consultation. Assuming a moderate effect size (Cohen’s d = 0.4), alpha error of 0.05, and statistical power of 80%, the required sample size was calculated as 65 participants. Considering 20% incomplete responses, a target of 80 participants was planned to improve the robustness of the analysis and allow for better subgroup evaluation.

Eighty participants seeking esthetic dental treatment were included in the study based on predefined inclusion and exclusion criteria. Patients aged between 18 and 60 years who presented with concerns related to smile esthetics and required procedures such as veneers, crowns, bleaching, or direct composite restorations were considered eligible for participation. Patients with severe periodontal disease, craniofacial anomalies, or an unwillingness to participate in the study were excluded.

During the initial appointment, each participant underwent a comprehensive clinical examination and a routine diagnostic evaluation. The proposed treatment plan was first explained to the patient using the conventional consultation method, which involved a verbal explanation of the treatment procedures and the expected outcomes based on clinical findings. Following the clinical examination and treatment planning, a digital smile simulation was generated for each patient using Smile Designer Pro software (Tasty Tech Ltd., London, United Kingdom). This simulation integrated facial and intraoral photographs to create a dynamic preview of the proposed esthetic outcome. The simulation was presented to the patient, allowing visualization of the anticipated result. Patient understanding of the treatment plan and confidence in the proposed treatment were assessed using a 5‑point Likert scale (1 = strongly disagree to 5 = strongly agree), both before and after viewing the simulation, enabling quantification of the impact of digital visualization on patient perception [7].

Patient satisfaction was assessed using the Visual Analog Scale (VAS), which is a simple and widely used tool for measuring subjective experiences in clinical research [8]. The VAS consisted of a 10-cm horizontal line representing a continuum of satisfaction levels. The left end of the scale indicated “not satisfied at all” (score 0), while the right end represented “extremely satisfied” (score 10). The patients were instructed to mark a point on the line that best reflected their level of satisfaction. The distance from the starting point to the marked point was measured in millimeters and recorded as the satisfaction score. The VAS was used to evaluate several parameters, including understanding of the treatment plan, ability to visualize the final esthetic outcome, confidence in the proposed treatment, satisfaction with dentist-patient communication, and overall satisfaction with the consultation process.

Following the initial assessment, a digital smile simulation was performed using Smile Designer Pro software (Tasty Tech Ltd., London, United Kingdom). The software was used to digitally simulate modifications in the tooth shape, alignment, and smile proportions based on the patient’s clinical presentation and esthetic requirements. The simulated smile design was then presented to the patient during the consultation to help visualize the expected treatment outcome and improve the understanding of the proposed esthetic changes.

After viewing the digital smile simulation, patients were again asked to evaluate their perception and satisfaction using the VAS for the same parameters assessed during the conventional consultation. This allowed the comparison of patient satisfaction before and after the DSD consultation, thereby assessing the impact of digital visualization on patient perception and confidence in the treatment plan.

Data were entered into a spreadsheet and analyzed using IBM SPSS Statistics for Windows, Version 20 (Released 2015; IBM Corp., Armonk, New York). Descriptive statistics are expressed as mean ± standard deviation for continuous variables and frequency with percentage for categorical variables. Patient understanding and confidence scores before and after the DSD consultation were compared using a paired t-test. The effect size was calculated using Cohen’s d to determine the magnitude of change. For overall satisfaction variables, the median and interquartile range (IQR) were also reported. Statistical significance was set at p < 0.05.

## Results

Eighty participants were included in the final analysis. Demographic and baseline characteristics of the study population are presented in Table 1. The majority of participants belonged to the 31-50 years age group, followed by the 18-30 years and >50 years age groups. Females constituted a greater proportion of the sample than males. Esthetic concerns were the most common reason for consultation, while 14 (17.5%) participants sought treatment primarily for functional reasons, and 18 participants (22.5%) reported both functional and esthetic concerns. Most participants had undergone previous dental treatment, whereas 18 (22.5%) reported no prior dental treatment. Regarding educational status, most participants had a high education level, followed by a medium and low education level. In addition, 24 (30%) participants reported previous cosmetic dental treatments.

**Table 1:** Demographic and baseline characteristics of the study participants (n = 80). Data are presented as frequency (n) and percentage (%).

Characteristic	Category	Frequency n (%)
Age group (years)	18-30	20 (25.0)
	31-50	42 (52.5)
	>50	18 (22.5)
Sex	Male	26 (32.5)
	Female	54 (67.5)
Reason for consultation	Esthetics	48 (60.0)
	Function	14 (17.5)
	Both	18 (22.5)
Previous dental treatment	Yes	62 (77.5)
	No	18 (22.5)
Education level	Primary	10 (12.5)
	Secondary	28 (35.0)
	Higher	42 (52.5)
Previous cosmetic dental treatment	Yes	24 (30.0)
	No	56 (70.0)
Socioeconomic status	Lower	12 (15.0)
	Middle	44 (55.0)
	Upper	24 (30.0)

Patient understanding of the proposed treatment plan before and after the DSD consultation is summarized in Table 2. A statistically significant improvement was observed in all the assessed parameters following DSD-assisted consultation. The mean score for clarity of procedures increased from 2.8 ± 0.9 to 4.5 ± 0.6, while understanding of treatment duration improved from 3.1 ± 1.0 to 4.3 ± 0.7. Similarly, significant improvements were noted in understanding of treatment costs (3.0 ± 1.1 to 4.2 ± 0.8) and understanding of treatment limitations (2.5 ± 0.9 to 4.0 ± 0.8). The overall understanding score also increased significantly from 2.9 ± 0.9 before consultation to 4.4 ± 0.6 after consultation. All comparisons demonstrated statistically significant differences (p < 0.001), indicating that the use of a DSD substantially enhanced patient understanding of the proposed treatment plan.

**Table 2:** Patient understanding of the proposed treatment plan before and after Digital Smile Design (DSD) consultation. Data are presented as mean ± standard deviation (SD), scores were measured using a 5-point Likert scale (1 = Not at all clear, 5 = Extremely clear), comparisons were performed using the paired t-test, and p < 0.05 was considered statistically significant. DSD: Digital Smile Design; SD: standard deviation.

Parameter (Understanding)	Pre-consultation mean ± SD	Post-consultation mean ± SD	Mean difference	t-value	p-value	Effect size (Cohen's d)
Clarity of procedures	2.8 ± 0.9	4.5 ± 0.6	1.7	14.2	< 0.001*	2.21
Understanding of treatment duration	3.1 ± 1.0	4.3 ± 0.7	1.2	9.8	< 0.001*	1.38
Understanding of costs	3.0 ± 1.1	4.2 ± 0.8	1.2	8.9	< 0.001*	1.24
Understanding of limitations	2.5 ± 0.9	4.0 ± 0.8	1.5	12.4	< 0.001*	1.76
Overall understanding	2.9 ± 0.9	4.4 ± 0.6	1.5	13.8	< 0.001*	1.96

Changes in patient confidence following the digital smile simulation are presented in Table 3. A significant increase in confidence level was observed after the DSD-assisted consultation. The mean score for confidence in the expected final smile outcome increased from 3.0 ± 1.1 to 4.6 ± 0.5, while confidence in the dentist’s treatment plan improved from 3.8 ± 0.9 to 4.7 ± 0.4. Similarly, the certainty to proceed with treatment increased from 3.2 ± 1.2 to 4.4 ± 0.7, and the ability to visualize the final result improved markedly from 2.5 ± 1.0 to 4.8 ± 0.3. All differences between the pre- and post-simulation scores were statistically significant (p < 0.001), demonstrating that the digital smile simulation significantly improved patient confidence in the proposed treatment.

**Table 3:** Changes in patient confidence following DSD. Data are presented as mean ± SD, scores were measured using a 5-point Likert scale (1 = Not at all clear, 5 = Extremely clear), comparisons were performed using the paired t-test, and p < 0.05 was considered statistically significant. DSD: Digital Smile Simulation; SD: standard deviation.

Parameter (Confidence)	Pre-simulation mean ± SD	Post-simulation mean ± SD	Mean difference	t-value	p-value	Effect size (Cohen's d)
Confidence in final smile	3.0 ± 1.1	4.6 ± 0.5	1.6	14.6	< 0.001*	3.07
Confidence in a dentist	3.8 ± 0.9	4.7 ± 0.4	0.9	9.8	< 0.001*	1.86
Certainty to proceed	3.2 ± 1.2	4.4 ± 0.7	1.2	11.2	< 0.001*	1.21
Visualization of the result	2.5 ± 1.0	4.8 ± 0.3	2.3	21.4	< 0.001*	1.27

Patient satisfaction with the DSD-assisted consultation process is summarized in Table 4. A high proportion of the participants reported positive responses regarding the effectiveness of DSD consultations. Most patients indicated that the simulation improved communication with the dentist (95%) and that the consultation process was easy to understand (97.5%). Additionally, 91.3% of the participants reported that the digital preview increased their excitement about the treatment, while 88.8% felt more involved in the treatment planning process. Overall satisfaction with the consultation was reported by 96.3% of the participants, and 98.8% indicated that they would recommend the DSD consultation approach to others.

**Table 4:** Overall patient satisfaction with the DSD consultation process. Satisfaction scores were assessed using a Visual Analog Scale (VAS) ranging from 1 to 10, where 1 = extremely dissatisfied/strongly disagree and 10 = extremely satisfied/strongly agree; interquartile range (IQR) = Q1–Q3, representing the middle 50% of responses; Agree/Strongly agree (%) represents the percentage of participants scoring 8–10 on the VAS scale. DSD: Digital Smile Design; SD: standard deviation; VAS: Visual Analog Scale; IQR: interquartile range.

Satisfaction statement	Mean ± SD	Median (IQR)	Agree/Strongly agree (%)
Simulation helped communication	9.2 ± 0.9	9.0 (9.0-10.0)	95%
Easy-to-understand consultation	9.4 ± 0.7	10.0 (9.0-10.0)	97.5%
Digital preview increased excitement	8.9 ± 1.1	9.0 (8.0-10.0)	91.3%
Felt involved in planning	8.7 ± 1.2	9.0 (8.0-9.0)	88.8%
Overall satisfaction	9.3 ± 0.8	9.5 (9.0-10.0)	96.3%
Would recommend to others	9.5 ± 0.6	10.0 (9.0-10.0)	98.8%

**Figure 1:**
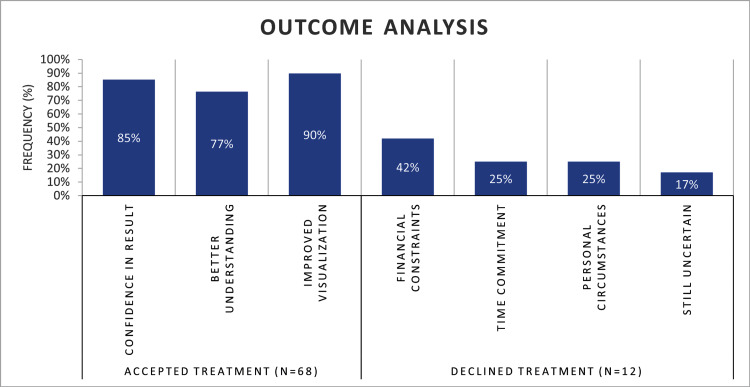
Treatment acceptance following Digital Smile Design consultation and reasons for acceptance among study participants.

The influence of the DSD on treatment acceptance is shown in Figure 1. The majority of patients accepted the proposed treatment following DSD consultation, whereas 12 participants (15%) chose not to proceed with treatment. Among those who accepted treatment, the most frequently reported reasons included improved confidence in the expected treatment outcome, better understanding of the treatment process, and enhanced visualization of the anticipated smile outcome. These findings suggest that incorporating DSD into the consultation process positively influences patients' decision-making and treatment acceptance.

## Discussion

This clinical study evaluated the impact of DSD on patient understanding, confidence, satisfaction, and treatment acceptance during esthetic dental treatment planning. The findings demonstrated significant improvements in all the assessed parameters following DSD-assisted consultation. The patients reported greater clarity regarding the treatment plan, improved confidence in the proposed outcomes, and high levels of overall satisfaction. Additionally, a substantial proportion of the participants accepted the recommended treatment after visualizing the simulated smile outcome, indicating that digital visualization plays a meaningful role in patient-centered esthetic dentistry.

One of the key findings of this study was the significant improvement in patients’ understanding of the proposed treatment plan after DSD consultation. Parameters such as the clarity of procedures, understanding of treatment duration, costs, and limitations showed statistically significant improvements. These findings suggest that digital visualization enhances communication between the clinician and patient by translating complex treatment concepts into easily understandable visual representations [9,10]. Traditional consultations often rely on verbal explanations and static images, which may not fully convey the expected esthetic outcomes. In contrast, digital smile simulation provides a visual preview of the potential treatment results, allowing patients to better comprehend the planned procedures [11]. Similar observations have been reported in previous studies, which have shown that DSD enhances treatment communication and facilitates better patient education during esthetic treatment planning [11-13]. Improved understanding can also help to reduce patient anxiety and uncertainty regarding treatment outcomes, thereby strengthening the decision-making process.

Another important observation in the present study was the significant increase in patient confidence after viewing a digital smile simulation. Confidence in the expected final smile, confidence in the dentist, certainty to proceed with treatment, and the ability to visualize the anticipated outcome improved significantly following DSD-assisted consultation. These findings support the concept that visual simulation helps to bridge the gap between clinical planning and patient expectations. When patients can clearly visualize the anticipated treatment outcome, they are more likely to trust the treatment plan and feel reassured regarding the procedure. Previous research in digital dentistry has similarly indicated that DSD enhances patient engagement and improves confidence in esthetic treatment outcomes [11,14,15]. The ability to preview potential results allows patients to actively participate in treatment planning, which may lead to improved satisfaction and stronger patient-clinician relationships.

The results of the present study also demonstrate high levels of overall patient satisfaction with the DSD consultation process. Most participants reported that the digital simulation improved communication with the dentist, made the consultation easier to understand, and increased their enthusiasm for treatment. Furthermore, a large proportion of patients reported feeling more involved in the treatment-planning process. These findings highlight the importance of patient-centered communication in modern dental practice [11,15]. Digital tools such as DSD not only assist clinicians in designing esthetic outcomes but also empower patients by allowing them to visualize and discuss potential treatment options. Recent evidence further supports the role of digital technologies in esthetic treatment planning. A systematic review and meta-analysis by Saini et al. reported that artificial intelligence-based DSD significantly improved both patient and clinician satisfaction while enhancing facial esthetic outcomes, highlighting the growing importance of digital tools in modern esthetic dentistry [16].

Another noteworthy finding was the high treatment acceptance rate observed after DSD consultation. In the present study, the majority of patients chose to proceed with the proposed treatment after viewing a digital smile simulation. The most frequently reported reasons included improved confidence in the expected outcome, better understanding of the treatment process, and enhanced visualization of the final smile. These results suggest that DSD positively influences patient decision-making. When patients clearly understand the anticipated esthetic results, they may feel more comfortable committing to treatment. Earlier studies evaluating digital treatment planning have reported similar findings, indicating that visual simulation can increase treatment acceptance and reduce hesitation associated with esthetic procedures [11,12,15]. This highlights the role of digital technology not only in treatment planning but also in improving patient motivation and engagement [4].

From a clinical perspective, the integration of DSD into routine esthetic dental practice offers several advantages. DSD facilitates more predictable treatment planning by allowing clinicians to systematically analyze facial proportions, tooth morphology, and smile dynamics. At the same time, it improves communication between the dentist and patient, which is essential for achieving satisfactory esthetic outcomes. The use of digital simulations may also reduce misunderstandings related to treatment expectations and enhance patient trust. Consequently, incorporating DSD into clinical workflows may contribute to improved treatment acceptance, enhanced patient satisfaction, and more predictable esthetic outcomes.

The present study has certain limitations that should be considered while interpreting the findings. First, this study was conducted at a single center using a single DSD software, which may limit the generalizability of the results to other clinical settings and digital platforms. Variations in software capabilities and clinician experience may influence the outcomes of digital smile simulations. Second, the study population demonstrated a relatively skewed demographic distribution, with a higher proportion of female participants and individuals with higher educational status, which may affect the external validity of the findings. Patient perception, expectations, and decision-making in esthetic dentistry can vary across different demographic and socioeconomic groups. Third, although a high treatment acceptance rate was observed, the reasons for treatment refusal among the 15% of participants who declined treatment were not specifically analyzed. Factors such as financial constraints, personal preferences, psychological factors, or differing esthetic expectations may have influenced this decision, and their evaluation could provide additional insights. Additionally, the study did not perform subgroup or correlational analysis to assess the influence of demographic variables such as age, gender, and socioeconomic status on patient satisfaction, confidence, or treatment acceptance. Future studies incorporating such analyses may help identify predictors of patient decision-making. Finally, this study assessed immediate patient-reported outcomes following the DSD consultation without evaluating long-term satisfaction after completion of treatment. Longitudinal studies are required to determine the sustained impact of DSD on patient satisfaction and clinical outcomes.

## Conclusions

Within the limitations of the present study, the use of DSD significantly improved patients’ understanding of the proposed treatment plan, confidence in the anticipated outcomes, and overall satisfaction during the consultation process. Visualization of the expected smile outcome facilitated better communication between the clinician and the patient and positively influenced treatment acceptance. These findings suggest that incorporating DSD into routine esthetic dental consultations can enhance patient engagement, improve decision-making, and contribute to more predictable and patient-centered treatment planning in contemporary esthetic dentistry.
